# Interpersonal Characteristics and Binge Eating among Patients Pursuing Bariatric Surgery

**DOI:** 10.3390/healthcare11212836

**Published:** 2023-10-27

**Authors:** Rawan Salameh-Dakwar, Roni Elran-Barak, Yara Zahra-Zeitoun, Gidon Soroka, Dvir Froylich, Ahmad Assalia, Yael Latzer

**Affiliations:** 1 School of Public Health, University of Haifa, Haifa 3498838, Israel; 2 Bnai Zion Medical Center, Haifa 31048, Israel; 3 Carmel Medical Center, Haifa 3436212, Israel; 4 Rambam Medical Center, Haifa 3109601, Israel

**Keywords:** obesity, bariatric surgery, binge eating, interpersonal, aggressiveness, self-esteem

## Abstract

Background: Preoperative binge eating behavior has been associated with difficulties in weight loss maintenance among patients pursuing bariatric surgery. However, limited data exists on the relationship between interpersonal difficulties and binge eating. Objectives: To identify interpersonal factors linked with binge eating among bariatric surgery candidates. Setting: One hundred and seventeen adult bariatric surgery candidates (BMI = 42.2 ± 5.2) from three different hospitals completed questionnaires on the day of their bariatric committee meeting for operation approval. Methods: Binge eating was assessed using the Questionnaire on Eating and Weight Patterns-5 (QEWP-5) as a dichotomous variable. Self-esteem was measured using the Rosenberg Self-Esteem Scale (RSES), and interpersonal characteristics were evaluated using the short version of the Inventory of Interpersonal Problems (IIP-32). Sociodemographic variables (age, gender, income, education) and BMI were considered as confounders. Results: Approximately 25% of bariatric surgery candidates reported experiencing binge eating episodes within the previous three months. Participants with binge eating exhibited significantly lower self-esteem and more interpersonal difficulties, particularly in the domains of aggressiveness and dependence, compared to those without binge eating. Logistic regression analysis revealed that aggressiveness was a significant predictor of binge eating in this sample. Conclusions: This study is the first, to the best of our knowledge, to investigate the relationship between interpersonal difficulties and binge eating among bariatric surgery candidates. The findings highlight the significant contribution of aggressiveness to binge eating and emphasize the importance of clinicians assessing patients’ interpersonal functioning, particularly with regard to aggressiveness, as a factor that may contribute to the maintenance and occurrence of binge eating behaviors.

## 1. Introduction

Binge eating, defined as overeating with a loss of control, is a core diagnostic criterion of binge eating disorder (BED) [1,2,3] and is linked with physical comorbidities such as diabetes and metabolic syndrome [4] and impaired emotional health [5]. Patients experiencing binge eating episodes are at risk for psychological difficulties such as distorted body image, depression, anxiety, impulsivity, and low self-esteem [6,7,8,9]. Although it is known that binge eating occurs in individuals across the weight spectrum, its prevalence increases with body mass index (BMI). Specifically, among bariatric surgery candidates (BMI > 40 or BMI > 35 with physical comorbidities), binge eating is highly prevalent [10]. About 2% to 53% of individuals who seek bariatric surgery have been reported to meet the DSM-5 criteria for BED (i.e., at least one weekly binge eating episode for three months) compared to 2–3% among the general population [11,12]. It is important to study the correlates of binge eating among patients seeking bariatric surgery [13], as the literature suggests that binge eating often persists after surgery and could affect post-surgery weight loss and its maintenance [14].

Interpersonal problems, or difficulties relating to others, are one of the most important causes of emotional distress [15] and are frequently present among patients with various mental disorders, such as eating disorders. A meta-analysis investigating the interpersonal difficulties of individuals with obesity found greater interpersonal stress among this population [16], but it is still unclear whether interpersonal difficulties cause obesity (e.g., through the use of binge eating as an escape from interpersonal difficulties) or whether obesity promotes interpersonal difficulties (e.g., as a result of weight stigma internalization). Although it has been found in some studies that individuals with obesity may be socially remote, cold, and distant in interpersonal relationships [16,17,18], other studies mapping the interpersonal profiles of people with obesity [17] have suggested that although these individuals tend to report elevated interpersonal distress, they are not homogeneous with regard to interpersonal problems. Given that studies have emphasized that binge eating, and not obesity per se, is linked with psychopathology among individuals seeking bariatric surgery, it is important to study the link between interpersonal difficulties and binge eating among this population [15]. Moreover, considering that low self-esteem has been established as a fundamental component of eating disorder symptomatology, including binge eating [19], and given the positive association between low self-esteem and interpersonal challenges, our study aims to explore the impact of interpersonal difficulties on binge eating, above and beyond low self-esteem, within the context of bariatric surgery candidates.

Bariatric surgery has been demonstrated to be an effective treatment for severe obesity, improving quality of life and decreasing mortality [20]. Yet significant weight loss after surgery is not guaranteed, and there may even be a recurrence of obesity due to (among other things) the existence of binge eating before and, particularly, after surgery [21,22]. To date no studies have examined the associations between interpersonal difficulties and binge eating among individuals seeking bariatric surgery. When comparing patients pursuing bariatric surgery who report binge eating with those who do not report binge eating, Riener and colleagues [14] found that patients with binge eating reported higher scores of disinhibited eating, unsatisfactory relationships, and an earlier onset of obesity, usually during childhood. One prior study [15], which compared patients with BED to non-BED patients with obesity, suggested that BED (and not obesity) was significantly linked with interpersonal difficulties.

Thus, the aim of this study was to understand whether specific interpersonal difficulties would be linked with binge eating among bariatric surgery candidates. The findings of the current study will help us identify correlates of binge eating among patients pursuing bariatric surgery, in order to improve behavioral treatments (e.g., cognitive behavioral therapy or interpersonal therapy [23]) for these patients, potentially influencing surgery outcomes.

## 2. Methods

### 2.1. Participants and Procedure

The study sample comprised a convenience sample of individuals seeking bariatric surgery at three hospitals in northern Israel between 2019 and 2022. The sample included 117 adults, between 18–70 years of age (M = 38.9, SD = 11.9), with a BMI > 35 (M = 42.2, SD = 5.2). The only inclusion criteria were bariatric surgery candidates who were able to read and write in Hebrew/Arabic. On the day of the bariatric surgery committee meeting, an explanation about the research was given by the researchers. Participants who met the inclusion criteria and agreed to answer the questionnaire were included (totaling about 90% compliance). Participants were granted full confidentiality. The study was approved by the Helsinki (ethics) Committee of the three hospitals and by the University of Haifa’s IRB (Institutional Review Board).

### 2.2. Measures

All questionnaires were taken from prior studies as explained below. If no validated Hebrew/Arabic version existed, we translated the questionnaire in accordance with the back translation method.

Binge eating. The Questionnaire on Eating and Weight Patterns-5 (QEWP-5) was used [24]. Participants who gave a “yes” answer to the following two questions were considered as individuals experiencing binge eating: “During the past three months, did you ever eat, in a short period of time (for example, a two-hour period) what most people would consider an unusually large amount of food?” (yes/no) and “During the times when you ate an unusually large amount of food, did you feel you could not stop eating or control what or how much you were eating?” (yes/no) [24]. This measure has been previously used among individuals seeking bariatric surgery (De Zwaan et al., 2003 [25]; Goodpaster, 2017 [26]).

Interpersonal characteristics. The Inventory of Interpersonal Problems—Short Version (the IIP-32) is a 32-item, self-reported measure of interpersonal difficulties [27]. Such difficulties can be experienced as things that people find “too hard” to do (e.g., join groups) or things that they do “too much” of (e.g., get irritated) [28]. The participants ranked the level of their distress regarding [29] difficulties on a Likert scale ranging from not at all (0) to extremely (4). The questionnaire consists of eight subscales [28]. Eight subscales and a total score were calculated in a similar way to how they were calculated in prior studies among patients pursuing bariatric surgery [30]. Higher scores point to more severe interpersonal difficulties. The Cronbach’s alphas for each subscale were as follows: not sociable (0.90), not assertive (0.83), not involved (0.78), not supportive (0.81), too open (0.79), too caring (0.73), too aggressive (0.86), and too dependent (0.68).

Self-Esteem. The Rosenberg 10-item Self-Esteem Scale (RSES) was used (e.g., “I feel I do not have much to be proud of” or “I wish I could have more respect for myself”). Each item is rated on a 4-point Likert scale ranging from strongly agree (1) to strongly disagree (4). Negatively worded items were reverse coded, so that higher scores on all RSES items indicated higher positive self-esteem [31]. The Cronbach’s alpha in the current study was 0.841. Self-esteem was defined as a control variable in this study, given its significant associations with binge eating on the one hand and interpersonal difficulties on the other, and given the fact that self-esteem has proven to be a core risk factor for eating disorder symptomatology [32], including binge eating (Pennesi & Wade, 2016 [29]). In addition, self-esteem may be related to interpersonal difficulties and socialization problems among patients with eating pathologies [19].

### 2.3. Data Analysis

The data were analyzed using SAS version 9.4, and *p* < 0.05 was considered significant. Categorical data were reported as the number (%) and were compared using the chi-square test. Continuous variables were reported as mean ± SD, and between-group comparisons were performed using a *t*-test or Wilcoxon two-sample test. A multiple logistic regression analysis was used for the prediction of binge eating. Missing data were handled by excluding participants with incomplete responses from the analyses.

We used OpenEpi (https://www.openepi.com/ accessed on 1 October 2023) to calculate the sample size required for comparing means between two groups using the following parameters: a 95% confidence level, 80% power, and a 2.8 ratio of sample size (Group 2 = 84/Group 1 = 30). The mean self-esteem for group 1 was set to 3 (0.5) and for group 2 was set to 2.7 (0.5). This calculation ensured that we had adequate statistical power to detect a difference of 0.3 units in mean self-esteem between the two groups.

## 3. Results

Table 1 presents our participants. There were no differences between those who reported binge eating (N = 30) and those who did not (N = 84) in terms of background characteristics. Table 2 shows that participants who reported binge eating were significantly (*p* < 0.01) more aggressive and more dependent than participants who did not report binge eating. When all eight personality domains were summed, participants who reported binge eating had greater general interpersonal difficulties than those without. Regarding self-esteem, participants without binge eating reported having significantly higher self-esteem than those who experienced binge eating. Of note is the fact that the average IIP-32 scores of our participants were fairly similar (and qualitatively a bit higher), relative to scores reported in prior studies [28].

Table 3 demonstrates a multivariable model predicting binge eating among bariatric surgery candidates. Only interpersonal characteristics that were significant in the univariable examination were entered into the model. Gender (female vs. male), age, BMI, and education were not found to predict binge eating. Regarding interpersonal characteristics, aggressiveness was found to be a significant predictor of binge eating, whereas dependence was not a significant predictor (OR = 1.801 and 0.921, respectively, *p* < 0.05). Self-esteem was also found to significantly predict binge eating among bariatric surgery candidates (OR = 0.309, *p* < 0.05).

## 4. Discussion

Binge eating, defined as overeating with a loss of control, is a core diagnostic criterion of binge eating disorder (BED), which has an adverse impact on weight gain and on psychological and physical health [33,34]. To the best of our knowledge, the current study is the first to examine the interpersonal difficulties of patients with obesity who are seeking bariatric surgery, in the presence or absence of binge eating. Several findings emerged. First, no significant correlations were found between background characteristics (BMI, gender, ethnicity, income, education, etc.) and binge eating. Second, two interpersonal characteristics were linked with binge eating—namely, aggressiveness and dependence. Third, a multivariable model suggested that only aggressiveness predicted binge eating. These findings may help us better understand some of the maintenance factors behind binge eating behaviors among individuals who seek bariatric surgery.

In our study, about 25% of the bariatric surgery participants reported binge eating during the previous three months. This finding is consistent with findings from studies stating that up to 50% of individuals pursuing bariatric surgery meet the criteria for BED [11,12]. For example, a study among people with class 3 obesity [35] suggests that as many as 53.2% participants had a high risk of eating disorders at baseline (i.e., before receiving a multidisciplinary treatment). There was no significant difference in BMI between participants with or without binge eating, whereas previous studies have suggested a heightened prevalence of eating psychopathology (e.g., binge eating) among people with severe obesity, relative to people with a lower BMI [36,37,38]. These inconsistencies can likely be attributed to the fact that our sample included only individuals living with severe obesity, in contrast to prior studies, which included individuals across the weight spectrum. Our findings seem to confirm, therefore, that the prevalence of binge eating is similar among all individuals living with severe obesity, regardless of their BMI.

To the best of our knowledge, this study is the first to investigate the correlation between binge eating and interpersonal difficulties among bariatric surgery participants. Our findings suggest a significant link between interpersonal difficulties and binge eating. A previous study [39] investigating the relationship between interpersonal characteristics and binge eating among women with BED found that the impact of interpersonal characteristics on binge eating was significant when controlling for negative affect. In our sample, however, the impact was significant when controlling for self-esteem, emphasizing the important role that interpersonal difficulties play in binge eating, above and beyond emotional distress and low self-esteem [39]. Another prior study [15] found that, compared to normal weight and obese participants, those with BED reported higher levels of interpersonal problems on all circumplex dimensions (except the vindictiveness dimension). In contrast, a prior study [17] examining interpersonal problems among people with obesity who were seeking dietary and psychological treatment for their weight problems suggested no associations between binge eating and participants’ interpersonal cluster. Tasca et al. (2012) found that patients with BED had higher total scores on all of the IIP subscales, including the total IIP score, compared to a control sample of individuals without BED and without psychiatric disorders [40]. However, when comparing BED patients with psychiatric outpatients, the two groups had similar IIP scores across all domains [40]. Although most studies suggest a link between interpersonal difficulties and binge eating, there are a few discrepancies between findings that may be attributed to different study methods and designs. Future studies should use longitudinal designs to determine the nature of the impact of interpersonal difficulties on binge eating.

In the current study, when both aggressiveness and dependence were entered into the model, only aggressiveness was found to impact binge eating. This finding is not surprising, as prior studies have suggested that the emotion most often preceding a binge episode is anger [41,42]. The literature about aggressiveness and anger among patients with eating psychopathology [42,43] relies on the understanding that there may be a significant relationship between binge eating and difficulties in emotion regulation [44]. The trade-off theory proposes that, in the presence of distressing emotional states (e.g., aggressiveness), people with difficulties in emotion regulation and eating psychopathology use binge eating to replace the more aversive emotional state (e.g., aggressiveness) with a less aversive affective condition (e.g., guilt after binge eating) [45]. The escape from self-awareness model posits that binge eating can serve as a means of narrowing the focus of attention toward food (i.e., an immediate stimuli) in order to block out the emotional pain (e.g., the pain resulting from aggressiveness) that preceded the binge episode [45]. In contrast, self-sacrificing was reported by Tasca et al. [40] as an interpersonal problem linked with binge eating, and Brugnera et al. [15] highlighted vindictiveness in this context.

### Limitations

As far as we know, the current study is the first to investigate whether specific interpersonal difficulties are linked with binge eating among individuals seeking bariatric surgery. The results will shed light on improving treatment for these individuals by identifying maintenance factors for binge eating and informing treatment plans that target binge eating reduction among patients pursuing bariatric surgery. However, several limitations should be taken into consideration. First, this was a cross-sectional study; as such, no causal connections can be inferred. In addition, our study sample was small and not representative; the results must therefore be viewed accordingly (i.e., they cannot be generalized to other populations). Finally, the data in this study were self-reported, and study participants filled out the questionnaires on the day of the bariatric committee meeting. It is reasonable to assume that they were under stress while filling out the questionnaires, and therefore these reports are subject to reporting bias and respondent bias.

## 5. Conclusions

This study highlights the important role of aggressiveness in binge eating among patients pursuing bariatric surgery, and specifically the role of aggressiveness as an interpersonal characteristic associated with binge eating. Careful assessment of interpersonal functioning, including aggressiveness, is essential for clinicians treating eating behaviors in patients pursuing bariatric surgery. Future prospective studies could confirm whether targeting aggressiveness, for example through cognitive behavioral therapy, in the treatment plan improves eating behaviors and potentially influences surgery outcomes.

## Figures and Tables

**Table 1:** Descriptive statistics of the participants by presence of binge eating episodes during the previous three months.

		Overall (N = 117)		Without Binge Eating (N = 84)		With Binge Eating (N = 30)		*p* Value
Variable		N	%	N	%	N	%	
**Age (** **Mean ± STD** **)**		38.9 ± 11.9		40.3 ± 12.4		35.9 ± 9.6		0.0874
**Gender**	Male	19	17.6	15	19.5	4	13.8	0.4961
	Female	89	82.4	62	80.5	25	86.2	
**Family status**	Living alone	64	55.7	47	56.6	17	58.6	0.8518
	Living with a partner	51	44.4	36	43.4	12	41.4	
**Having kids**	No	35	30.4	23	28.0	9	30.0	0.7396
	Yes	80	69.6	59	72.0	21	70.0	
**Education**	Less than 12 years	17	15.7	13	17.1	4	13.8	0.7752
	More than 12 years	91	84.3	63	82.9	25	86.2	
**Employed**	No	77	68.8	55	67.9	19	68.9	0.9966
	Yes	35	31.3	26	32.1	9	32.1	
**Income level ^1^ (Mean ± STD, Median)**		5.5 ± 2.3, 5.0		5.6 ± 2.2, 5.0		5.3 ± 2.6, 5.0		0.5541
**BMI (Mean ± STD, Median)**		42.4 ± 5.2, 41.3		42.3 ± 5.3, 41.1		43.0 ± 5.2, 42.6		0.5003
**Ethnicity**	Jewish	56	47.9	39	46.4	15	50.0	0.7366
	Arab	61	52.1	45	53.6	15	50.0	

^1^ Income varies from 1 (<1440 NIS a month per household) to 10 (>24,000 NIS a month per household) NIS = New Israeli Shekel.

**Table 2:** The associations of binge eating with interpersonal characteristics and self-esteem.

	Without Binge Eating N = 84			With Binge Eating N = 30				
Variable	N	Mean	STD	N	Mean	STD	*p* Value	Cronbach’s Alpha
Hard to be sociable	84	1.4	1.1	29	1.9	1.1	0.0551	0.788
Hard to be assertive	84	1.2	0.9	29	1.6	1.0	0.0825	0.698
Hard to be supportive	84	1.4	1.0	29	1.6	0.9	0.2143	0.631
Hard to be involved	84	1.3	1.0	29	1.7	1.0	0.0701	0.564
Too aggressive	83	1.1	1.0	29	1.8	1.1	**0.0019**	0.781
Too open	84	1.9	0.8	29	1.9	0.9	0.8329	0.421
Too caring	84	1.7	1.0	29	2.0	0.9	0.1603	0.575
Too dependent	83	1.1	0.9	29	1.6	1.1	**0.0243**	0.744
Interpersonal total score (IIP-32)	84	1.3	0.6	29	1.7	0.7	**0.0035**	0.892
Self-esteem total score (RSES)	84	3.0	0.5	29	2.7	0.5	**0.0030**	0.841

Note: The Rosenberg Self-Esteem Scale (RSES); Inventory of Interpersonal Problems (IIP-32). Significant *p* values appear in bold.

**Table 3:** Logistic regression model predicting binge eating.

	OR	95% CI		*p*-Value
Gender (female vs. male)	0.808	0.193	3.378	0.7698
Age	0.986	0.940	1.034	0.5612
BMI	0.989	0.888	1.101	0.8416
Education (above 12 years vs. below 12 years)	0.983	0.198	4.880	0.9830
Ethnicity (Arab vs. Jewish)	0.816	0.282	2.361	0.7076
Too aggressive	1.802	1.036	3.136	**0.0371**
Too dependent	0.921	0.502	1.690	0.7903
Self-esteem	0.309	0.108	0.881	**0.0280**

Note: Significant *p* values appear in bold.

## Data Availability

The data is available upon request to the corresponding author.
